# Age-stratified respiratory viral detection patterns and exploratory environmental associations in a tertiary-care hospital testing cohort in Istanbul, Türkiye

**DOI:** 10.3389/fmicb.2026.1893118

**Published:** 2026-07-16

**Authors:** Murat Yaman, Arzu Ilki

**Affiliations:** 1 Medical Microbiology, Marmara University Pendik Training and Research Hospital, Istanbul, Türkiye; 2 Department of Medical Microbiology, Faculty of Medicine, Marmara University, Istanbul, Türkiye

**Keywords:** age-stratified analysis, air quality, meteorological exposures, molecular co-detection, multiplex RT-PCR, respiratory viral detection

## Abstract

Respiratory viral detection in large metropolitan clinical settings is shaped by age, seasonality, testing behavior, and environmental context, but these dimensions are rarely evaluated together using multiplex molecular data. Istanbul's transcontinental position, dense urban structure, and heterogeneous climatic and air-quality conditions provide a relevant setting for integrated analysis of respiratory viral detection. This retrospective sample-level study analyzed 21,151 clinician-requested respiratory specimens tested by multiplex reverse transcription polymerase chain reaction in Istanbul, Türkiye, during 2022–2025. The study aimed to characterize age-stratified respiratory viral detection patterns and to explore short-term associations with meteorological and pollutant-specific air-quality exposures. Detection patterns were classified as single-target, dual-target, or ≥3-target positivity. ERA5-derived meteorological variables and station-based pollutant data were linked to specimen collection dates using a predefined incubation-informed 1–9-day pre-sampling exposure window. Environmental analyses were exploratory and interpreted using effect estimates, 95% confidence intervals, nominal *p*-values, and false-discovery-rate-adjusted q values where applicable; SARS-CoV-2-specific analyses were restricted to 2024–2025. After excluding SARS-CoV-2, at least one evaluated respiratory viral target was detected in 4,836 specimens (22.9%). Positive specimens were obtained from younger patients than target-negative specimens [median age, 6 years (interquartile range, 1–32) vs. 19 years [interquartile range, 4–59]; *p* = 7.58 × 10^−146^]. Children aged 1–5 years accounted for 30.1% of single-target, 44.1% of dual-target, and 48.2% of ≥3-target detections. Enterovirus/rhinovirus was the most frequently detected viral target, identified in 1,604 of 4,836 positive specimens (33.2%). Seasonal patterns varied by age group and viral target. Exploratory exposure analyses showed crude signals linking low temperature and selected pollutant-specific exposures with dual+≥3-target positivity, with attenuation of most estimates after year–month temporal-control sensitivity analyses. Overall, respiratory viral detection in this tertiary-care hospital testing cohort varied mainly by age, viral target, and season, while the environmental analyses provided temporally controlled contextual signals rather than confirmatory causal evidence. These findings support cautious, context-aware interpretation of multiplex respiratory viral testing data in large urban clinical settings.

## 1 Introduction

Respiratory viral detection patterns and circulation dynamics are shaped by overlapping demographic, seasonal, environmental, immunological, and social factors. Age-related susceptibility, contact intensity, viral target distribution, molecular co-detection, and local environmental conditions all contribute to the heterogeneity observed in respiratory viral detection patterns (Piret and Boivin, 2022; He et al., 2023; Neumann and Kawaoka, 2022; Smits and Jochems, 2024). Accordingly, interpretation of respiratory viral testing data should move beyond overall positivity rates and incorporate age group, seasonality, target-specific detection, molecular co-detection, and environmental context within an integrated analytical framework.

Respiratory viral detections are often concentrated in children, partly because of close-contact environments such as daycare centers and schools and fewer previous encounters with common respiratory viruses (Smits and Jochems, 2024; Chen et al., 2020). In early life, respiratory syncytial virus (RSV), parainfluenza viruses, adenovirus, and enterovirus/rhinovirus are closely linked to age-specific susceptibility and contact patterns (Yaman et al., 2022; Krivonosov et al., 2024; Pierangeli et al., 2025). In older age groups, immunosenescence, comorbidities, and age-related risk gradients may influence the clinical consequences of respiratory viral disease, as was particularly evident during the SARS-CoV-2 pandemic (Channappanavar and Perlman, 2020; Poletti et al., 2021).

Seasonal patterns of respiratory viral activity differ by viral target, geography, climate, and population structure. In temperate settings, several respiratory viruses show winter-weighted activity, but the magnitude and timing of seasonal peaks are not uniform (Bloom-Feshbach et al., 2013; Xu et al., 2021; Price et al., 2019; Couto et al., 2025). Temperature, relative humidity, precipitation, and wind speed may contribute to these patterns by influencing viral stability, aerosol behavior, host airway defenses, indoor contact patterns, and healthcare-seeking behavior (Lowen et al., 2007; Li et al., 2011; Guarnieri et al., 2023; Huang et al., 2023). Studies from Türkiye and other settings have also reported associations between respiratory infections and meteorological indicators, including temperature, humidity, wind speed, and atmospheric pressure (Yüksel et al., 2024; Saadi et al., 2021).

Air quality represents another relevant environmental dimension in respiratory viral epidemiology. Particulate matter and gaseous pollutants may affect respiratory susceptibility through disruption of the airway epithelial barrier, oxidative stress, altered inflammatory responses, impaired mucociliary clearance, and modulation of antiviral defenses (Ciencewicki and Jaspers, 2007; Burbank, 2023; Poniedziałek et al., 2024). PM_2.5_, PM_10_, NO_2_, SO_2_, CO, and O_3_ are widely used air-quality indicators with established relevance for respiratory health (World Health Organization, 2021). Evidence from the COVID-19 and post-pandemic periods further suggests that relationships among air quality, meteorological conditions, and respiratory viral detection may be context specific rather than uniform across settings or viral targets (Orak, 2022; Matera et al., 2023; Mese et al., 2024; Chen et al., 2025; Li et al., 2025; Shi et al., 2025).

Istanbul provides an informative setting for evaluating respiratory viral detection patterns in relation to demographic and environmental conditions. As a densely populated transcontinental metropolis with high human mobility, marked seasonality, and spatially heterogeneous air-quality profiles, the city offers a relevant context for integrated analysis of respiratory viral detection. Previous studies from Istanbul and Türkiye have shown spatial, seasonal, and temporal variation in urban air quality, supporting the interpretation of environmental monitoring data alongside health-related outcomes (Menteşe and Ogurtani, 2022; Celik and Gul, 2022; Bostan et al., 2023; Dönmez and Aslan, 2023; Al-Rousan et al., 2025). In parallel, post-pandemic respiratory virus studies from Türkiye have highlighted altered detection dynamics and the importance of sustained molecular monitoring (Mese et al., 2024; Koçer et al., 2025).

In this study, we retrospectively analyzed 21,151 respiratory specimens tested with a multiplex respiratory viral panel as part of clinician-requested testing in Istanbul during 2022–2025. The study period was considered a transition and post-pandemic phase, during which respiratory viral detection patterns, testing practices, and SARS-CoV-2-related diagnostic workflows continued to evolve. This framing is consistent with recent studies that have distinguished the period beginning in 2022 from the earlier acute-pandemic phase when evaluating changes in respiratory virus detection and circulation dynamics (Koçer et al., 2025).

We aimed to characterize age- and season-stratified viral detection patterns, molecular co-detection profiles, and detection complexity. We also explored short-term associations between meteorological and pollutant-specific air-quality exposures and detection complexity using an incubation-informed 1–9-day pre-sampling exposure framework based on published incubation-period evidence for common respiratory viruses (Spencer et al., 2022; Gressani et al., 2025; Tsinaris et al., 2026). These analyses were intended to support age-stratified and environmentally informed interpretation of respiratory viral testing data in a large urban clinical setting, while keeping environmental associations exploratory rather than confirmatory.

## 2 Materials and methods

### 2.1 Study design and setting

This retrospective, laboratory-based, sample-level study was conducted at the Medical Microbiology Laboratory of Marmara University Pendik Training and Research Hospital, Istanbul, Türkiye. Respiratory specimens tested with a multiplex respiratory viral panel between January 1, 2022, and December 31, 2025, were included. The study characterized age-stratified respiratory viral detection patterns and explored their variation by season, meteorological exposures, and air-quality indicators. Because testing was clinician-requested, the findings represent the tested clinical population rather than population-level incidence.

### 2.2 Study population and respiratory specimens

A total of 21,151 respiratory specimens submitted for routine diagnostic testing were included. Specimens were obtained from patients for whom respiratory viral panel testing was requested by clinicians. Upper respiratory tract samples were collected as nasopharyngeal swabs and transported in vNAT^®^ transfer tubes containing 2 mL of transport medium (Bioeksen R&D Technologies, Istanbul, Türkiye). Samples were processed according to the manufacturer's recommendations.

Respiratory viral panel results were retrieved from the hospital Laboratory Information Management System and linked with age, sex, and specimen collection date. Records with missing, inconsistent, or unverified information were excluded during data quality control. Age was categorized as <1 year, 1–5 years, 6–17 years, 18–40 years, 41–64 years, and ≥65 years. Seasons were defined according to the Northern Hemisphere calendar: winter, December–February; spring, March–May; summer, June–August; and autumn, September–November.

### 2.3 Multiplex molecular respiratory viral testing

Respiratory viral targets were detected using the Bio-Speedy Respiratory Tract RT-PCR MX-24S Panel (Bioeksen R&D Technologies, Istanbul, Türkiye), performed as part of routine laboratory diagnostics. For multiplex reverse transcription polymerase chain reaction testing, 90 μL of patient sample from the vNAT tube was mixed with 90 μL of 2 × Prime Master Mix, yielding a total preparation volume of 180 μL. Amplification was performed on the CFX96 Real-Time PCR System (Bio-Rad, USA) according to the manufacturer's thermal cycling protocol.

Amplification curves and cycle threshold values were interpreted using the instrument software and manufacturer-recommended thresholds. The panel included SARS-CoV-2, influenza A virus (IAV), influenza B virus (IBV), seasonal human coronaviruses (HCoVs), human parainfluenza viruses (HPIVs), human metapneumovirus (hMPV), adenovirus (AdV), human bocavirus (HBoV), human enterovirus, human rhinovirus, human parechovirus (HPeV), and respiratory syncytial virus (RSV) A/B. Enterovirus and rhinovirus were analyzed together as enterovirus/rhinovirus (E-RV) because of the panel-level reporting structure. Thermal cycling conditions and the complete list of respiratory viral targets included in the multiplex panel are provided in Supplementary File 1 and Supplementary Tables 1, 2.

Positive results were interpreted as molecular detection of viral nucleic acid. Co-detection was defined as detection of more than one viral target in the same specimen and was not interpreted as confirmed clinical co-infection, viral viability, or causal attribution to symptoms.

### 2.4 Viral positivity and detection-pattern definitions

A specimen was classified as target-positive when at least one respiratory viral target was detected. For analytical consistency across the full study period, the main 4-year analysis excluded SARS-CoV-2 and was restricted to non-SARS-CoV-2 respiratory viral targets with comparable multiplex-panel coverage.

In the main non-SARS-CoV-2 analysis, target-positive specimens were classified according to the number of detected viral targets as single-target, dual-target, or ≥3-target positive. Dual-target and ≥3-target positivity were combined as dual+≥3-target positivity to represent molecular detection complexity. Combined dual+≥3-target positivity was prespecified as the primary environmental outcome. Overall non-SARS-CoV-2 target positivity, single-target positivity, dual-target positivity, and standalone ≥3-target positivity were retained as secondary or descriptive outcomes, depending on the analysis.

SARS-CoV-2-specific analyses were restricted to the 2024–2025 multiplex-panel period because SARS-CoV-2 testing in 2022–2023 was performed using different PCR workflows and was affected by national testing policies; therefore, these results were not uniformly captured in the Laboratory Information Management System. This restriction limited longitudinal interpretation for SARS-CoV-2 and may have led to under-ascertainment of SARS-CoV-2-associated co-detection in 2022–2023. Accordingly, SARS-CoV-2-specific results were interpreted as restricted sensitivity outputs rather than as part of the main 4-year comparative framework.

### 2.5 Environmental data integration and study area

Meteorological and air-quality variables were linked to respiratory viral panel results by specimen collection date. Istanbul was represented using central coordinates of 41.0082° N and 28.9784° E. For meteorological data extraction, the study area was bounded by latitudes 40.8–41.2° N and longitudes 28.8–29.2° E. Air-quality exposures were derived from daily measurements recorded at the Kartal urban monitoring station, as detailed in Section 2.7.

### 2.6 Meteorological exposure data

Meteorological data were obtained from the ERA5 reanalysis dataset provided by the European Centre for Medium-Range Weather Forecasts (Hersbach et al., 2020). Variables included ambient temperature, dew point temperature, 10-m zonal and meridional wind components, and total precipitation. Relative humidity was calculated from temperature and dew point temperature using the Magnus formula. Wind speed was derived from the 10-m wind components and converted to km/h, and total precipitation was converted from meters to millimeters.

Hourly ERA5 grid values within the predefined Istanbul bounding box were first aggregated to daily values before linkage to specimen collection dates. Temperature, relative humidity, and wind speed were summarized as daily means, whereas precipitation was summarized as daily cumulative precipitation. These daily values were then used to construct the predefined incubation-informed 1–9-day pre-sampling exposure window, excluding the day of sampling. The window was defined before reanalysis to reflect the expected incubation range of common respiratory viral targets and was informed by published incubation-period estimates, including Spencer et al. (2022), Gressani et al. (2025), and Tsinaris et al. (2026). Because symptom-onset dates were unavailable, exposure windows were anchored to specimen collection date.

For exposure-window analyses, temperature, relative humidity, and wind speed were averaged across days 1–9 before sampling, whereas precipitation was accumulated over the same period. Low temperature was defined as <10 °C, high relative humidity as ≥70%, and high wind speed and cumulative precipitation as values at or above the 75th percentile of the corresponding exposure distribution.

### 2.7 Air-quality exposure data

Daily air-quality indicators included PM_2.5_, PM_10_, NO_2_, SO_2_, CO, and O_3_. Hourly station-based data were obtained from the World Air Quality Index Project (2026) for the Kartal urban monitoring station in Istanbul and cross-checked against the national air-quality monitoring network of the Republic of Türkiye Ministry of Environment, Urbanization and Climate Change (2026). The Kartal station (station code: 0134029; 40.9110° N, 29.1830° E) was the single air-quality monitoring source used in the present analyses.

Hourly records were standardized for date-time formats, variable naming, and numeric format before daily aggregation. Physically impossible negative values were treated as missing. Daily pollutant averages were calculated only when at least 18 valid hourly measurements were available for a given pollutant and date. No numerical imputation was performed at the hourly, daily, or exposure-window level.

For each pollutant, exposure was summarized as the mean concentration across days 1–9 before specimen collection, excluding the collection day. High-exposure categories were defined as values at or above the 75th percentile of the corresponding pollutant distribution. A valid exposure-window summary required at least seven valid daily values within days 1–9; specimens with fewer valid preceding daily values were excluded only from the corresponding pollutant-specific analysis. Analyses were conducted separately for each pollutant rather than using a composite AQI-equivalent metric, thereby preserving pollutant-specific interpretability.

Environmental data sources, preprocessing rules, pollutant-specific daily completeness, exposure-window definitions, and exposure categories are summarized in Supplementary Data Sheet 1, Supplementary Table 3.

### 2.8 Statistical analysis

Data processing, modeling, and visualization were performed using IBM SPSS Statistics version 30.0 (IBM Corp., Armonk, NY, USA) and Python version 3.12 with Pandas, NumPy, SciPy, statsmodels, and Matplotlib.

Categorical variables were summarized as *n* (%) and compared using the chi-square test or Fisher's exact test, as appropriate. Skewed continuous variables, including age, were summarized as median (interquartile range, IQR) and compared using the Mann–Whitney U test.

For environmental analyses, combined dual+≥3-target positivity was used as the prespecified primary outcome, as defined in Section 2.4. The remaining positivity categories were retained as secondary or descriptive outcomes. Crude relative risks (RRs) with 95% confidence intervals were estimated for high-exposure vs. reference-category comparisons. Among non-SARS-CoV-2 target-positive specimens, differences in detection-pattern composition were expressed as odds ratios with 95% confidence intervals.

Year–month temporal-control analyses for combined dual+≥3-target positivity used stratified Poisson regression, with calendar year–month as the stratum. Exposure categories were compared within strata. Records without a valid exposure-window summary and strata lacking both exposure categories were excluded from the corresponding analysis. Exposure-specific sample sizes, event counts, informative strata, RRs, confidence intervals, nominal *p*-values, and FDR-adjusted q values are reported in Supplementary Data Sheet 3.

As a descriptive ecological sensitivity analysis, overall monthly non-SARS-CoV-2 viral positivity was correlated with monthly mean temperature using Spearman rank correlation. This analysis was not treated as primary environmental evidence because both measures may share seasonal and calendar-time structure.

All environmental analyses were exploratory. Benjamini–Hochberg correction was applied separately to crude and year–month temporal-control estimates within the four meteorological and six pollutant-specific comparisons, respectively. Interpretation emphasized effect magnitude, precision, temporal-control robustness, and FDR-adjusted evidence rather than nominal *p*-values alone. The sparse standalone ≥3-target category was not used as a primary inferential outcome and was reported descriptively or in supplementary analyses.

## 3 Results

### 3.1 Overall viral detection and cohort characteristics

**Figure 1:**
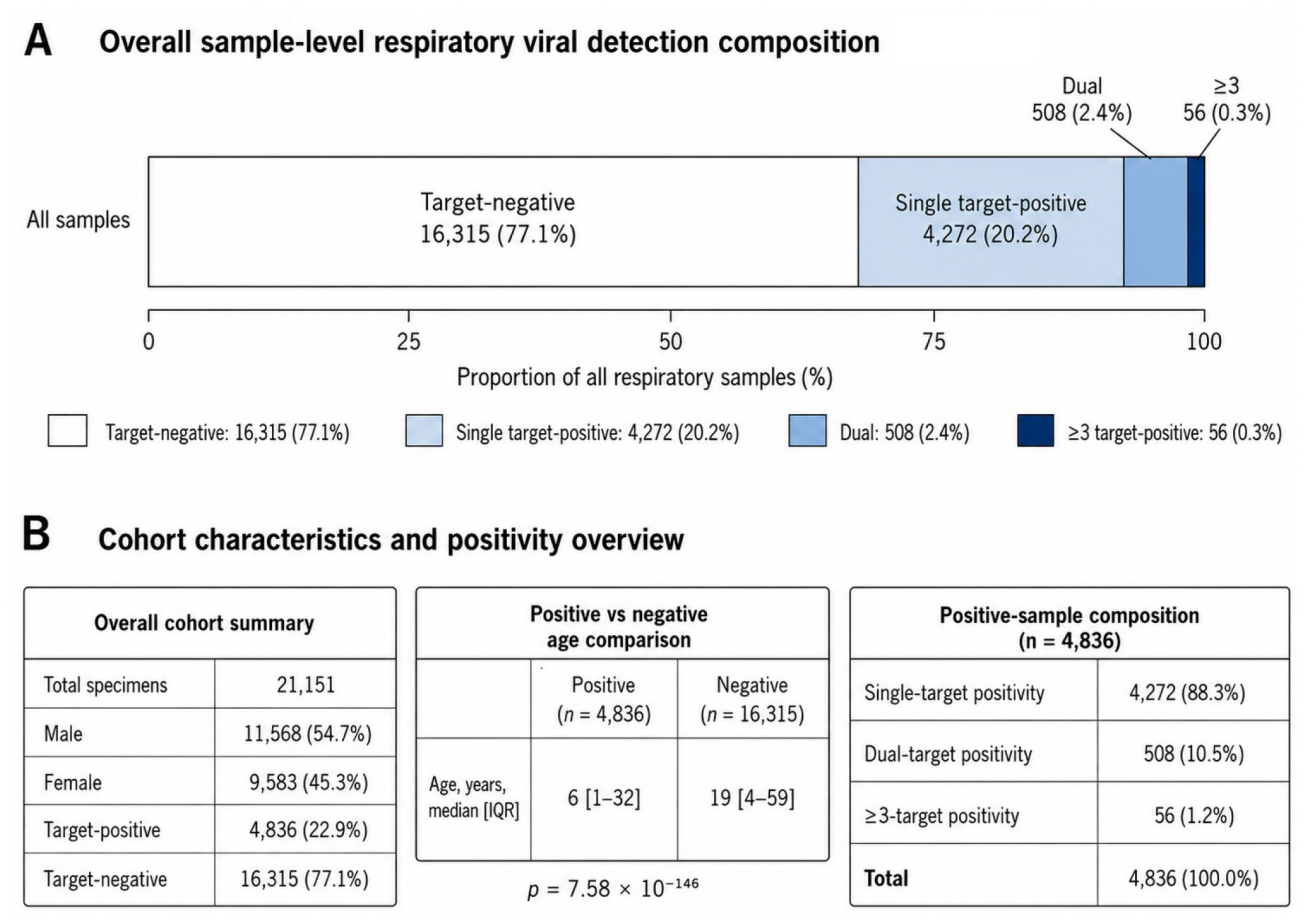
Sample-level non-SARS-CoV-2 respiratory viral detection composition and cohort overview. **(A)** Overall sample-level detection composition in the main 4-year analysis excluding SARS-CoV-2. Specimens were classified as non-SARS-CoV-2 target-negative, single-target positive, dual-target positive, or ≥3-target positive. Percentages in Panel A are calculated among all tested specimens. **(B)** Cohort characteristics, age comparison between target-positive and target-negative specimens, and positive-sample composition. Percentages for single-, dual-, and ≥3-target positivity in Panel B are calculated among non-SARS-CoV-2 target-positive specimens. Age is reported as median (IQR); the *p-*value refers to the Mann–Whitney U test. Values are *n* (%) unless otherwise indicated.

Between 1 January 2022 and 31 December 2025, 21,151 respiratory specimens tested using a multiplex respiratory viral panel were evaluated. In the main 4-year analysis, which focused on viral targets with comparable testing coverage, at least one evaluated non-SARS-CoV-2 viral target was detected in 4,836 specimens (22.9%), whereas 16,315 specimens (77.1%) were negative for all evaluated non-SARS-CoV-2 viral targets. The overall sample-level detection composition and cohort overview are shown in Figure 1.

Among all tested specimens, 4,272 (20.2%) showed single-target positivity, 508 (2.4%) showed dual-target positivity, and 56 (0.3%) showed ≥3-target positivity. Among non-SARS-CoV-2 target-positive specimens, the corresponding positive-sample composition was 88.3%, 10.5%, and 1.2%, respectively. The tested cohort included 11,568 male patients (54.7%) and 9,583 female patients (45.3%). Specimens positive for at least one evaluated non-SARS-CoV-2 viral target were obtained from younger patients than non-SARS-CoV-2 target-negative specimens [median age, 6 years (IQR, 1–32) vs. 19 years (IQR, 4–59); Mann–Whitney U test, *p* = 7.58 × 10^−146^].

**Figure 2:**
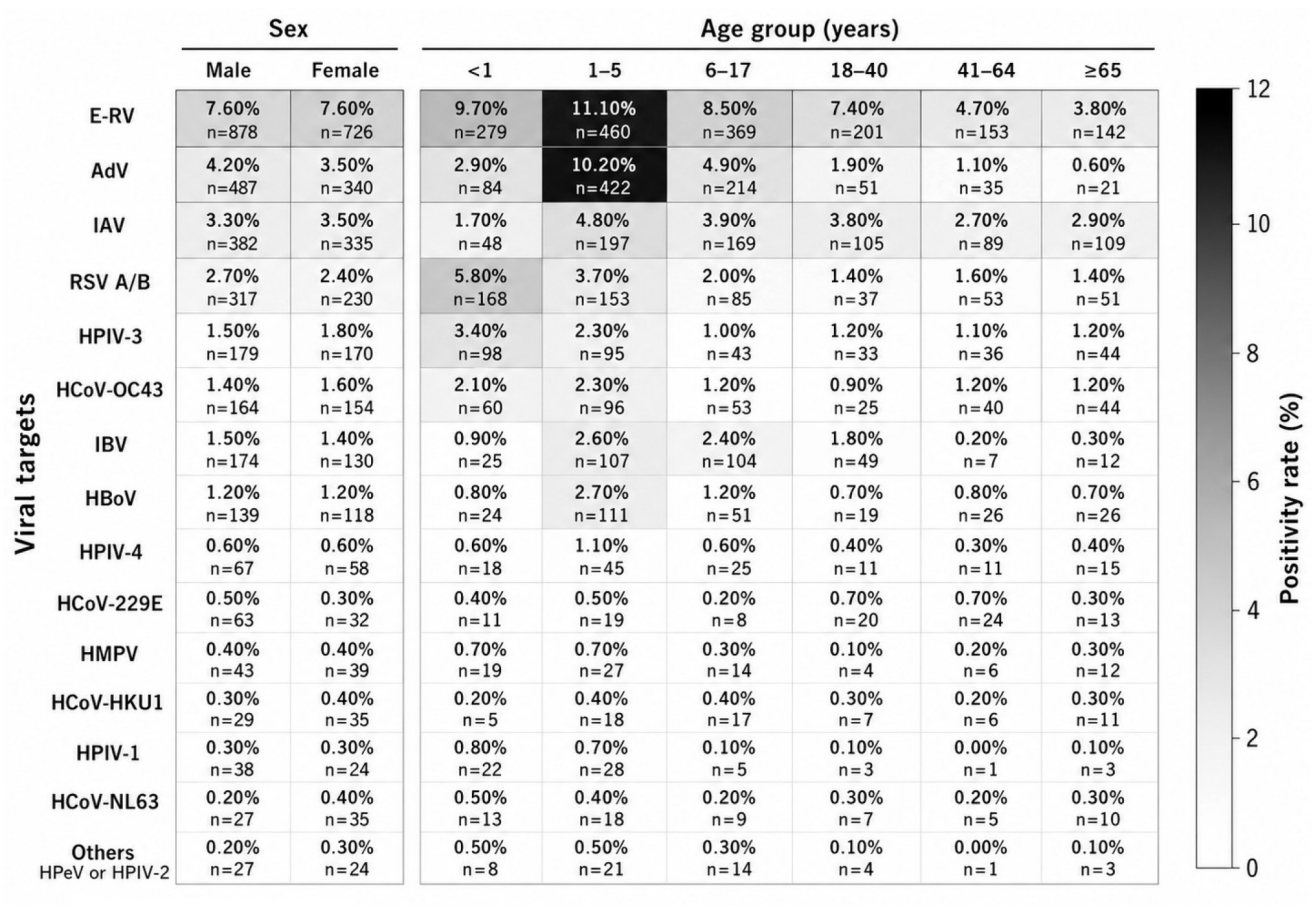
Target-specific non-SARS-CoV-2 respiratory viral positivity rates by sex and age group. Heatmap cells display target-specific positivity rates and positive counts for viral targets included in the main 4-year non-SARS-CoV-2 analysis. Percentages were calculated within each sex or age stratum and are shown to two decimal places; exact unrounded values were used for interpretation, and no cell was highlighted solely on the basis of rounded equality. Higher positivity rates for E-RV and AdV were observed in children aged 1–5 years, whereas RSV A/B and HPIV-3 were relatively more prominent among children aged <1 year. “Others” denotes HPeV and HPIV-2. Because co-detections were allowed, target-specific counts are not mutually exclusive.

Target-specific positivity rates by sex and age group are shown in Figure 2. No marked sex-specific difference was observed in the overall distribution of the main viral targets. By contrast, age-stratified patterns showed clear target-specific variation. E-RV was the most frequently detected viral target overall, whereas AdV and E-RV positivity were highest in the 1–5-year age group. RSV A/B and HPIV-3 were more prominent among children aged <1 year. Detailed age-stratified detection frequencies for individual respiratory viral targets are provided in Supplementary Data Sheet 1 and Supplementary Table 4.

### 3.2 Age-stratified detection patterns

**Figure 3:**
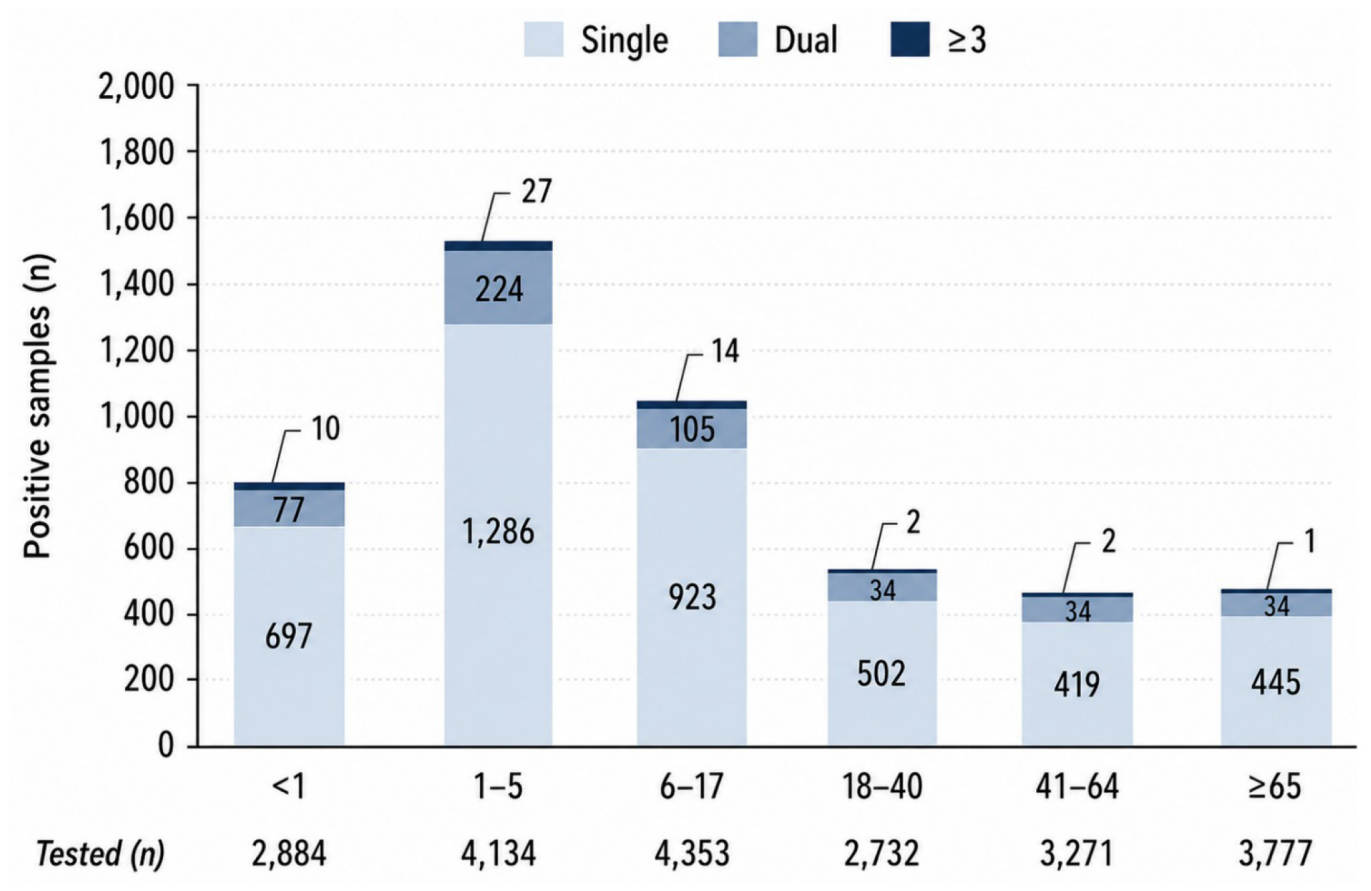
Age-stratified distribution of non-SARS-CoV-2 target-positive specimens by detection category. Stacked bars show specimens positive for at least one evaluated non-SARS-CoV-2 viral target in the main 4-year analysis, stratified by age group and detection category: single-target, dual-target, and ≥3-target positivity. Tested specimen counts are shown below each age group. Dual+≥3-target positivity was more frequent in pediatric age groups, particularly among children aged 1–5 years, whereas single-target positivity predominated across all age groups.

Age-stratified detection patterns are shown in Figure 3. Viral positivity in the main analysis was highest in the 1–5-year age group (1,537/4,134; 37.2%), followed by the <1-year age group (784/2,884; 27.2%) and the 6–17-year age group (1,042/4,353; 23.9%). Positivity was lower in adults: 538/2,732 (19.7%) among those aged 18–40 years, 455/3,271 (13.9%) among those aged 41–64 years, and 480/3,777 (12.7%) among those aged ≥65 years.

Single-target positivity predominated in all age groups. However, dual+≥3-target positivity was concentrated in pediatric strata, particularly among children aged 1–5 years. This group accounted for 30.1% of all single-target positive specimens (1,286/4,272), 44.1% of dual-target positive specimens (224/508), and 48.2% of ≥3-target positive specimens (27/56). The proportion of dual+≥3-target positivity among target-positive specimens was 16.3% in the 1–5-year group, compared with 6.7–7.9% across adult age groups. The standalone ≥3-target stratum was small (*n* = 56) and was therefore interpreted descriptively.

### 3.3 Respiratory viral co-detection patterns

**Figure 4:**
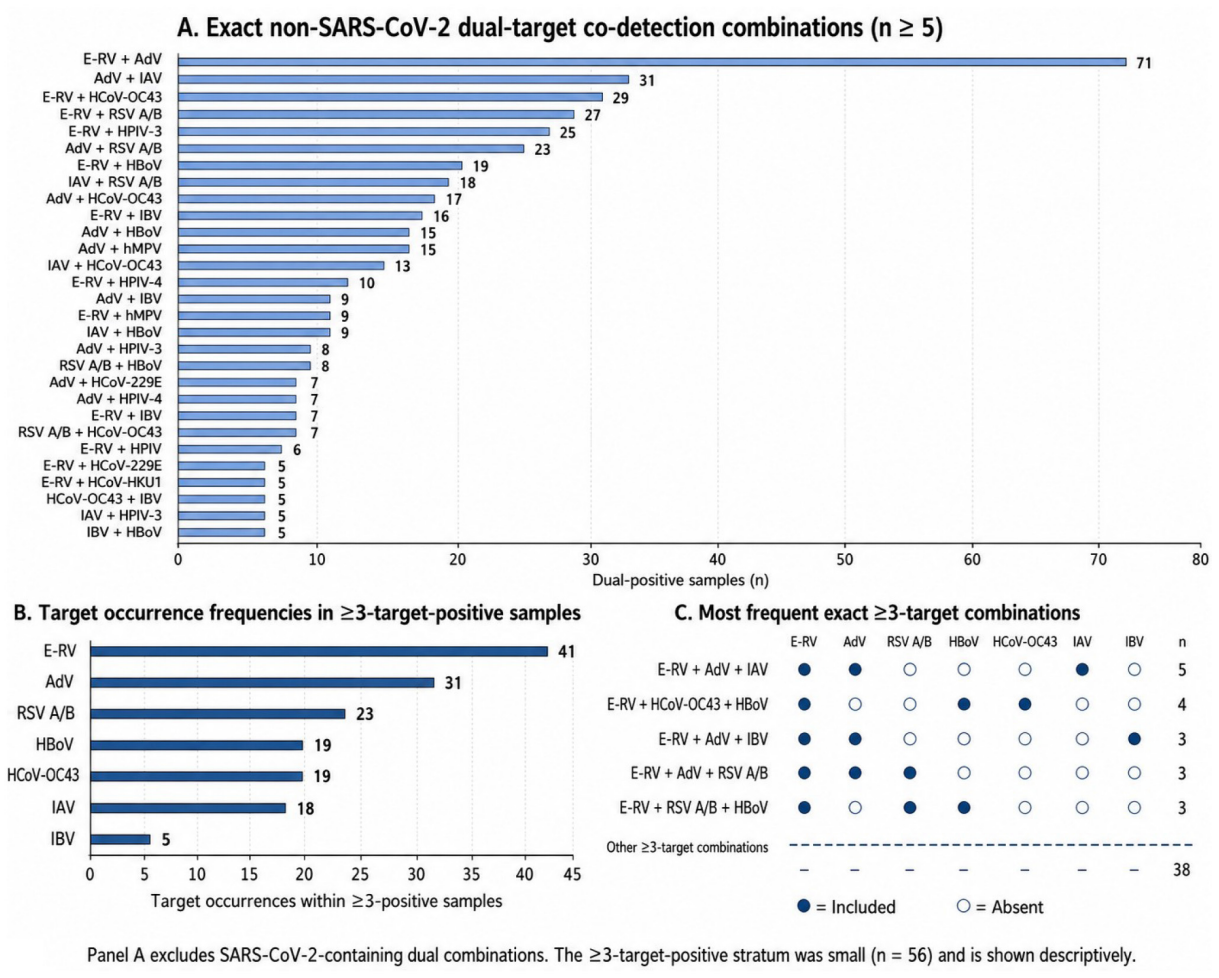
Respiratory viral molecular co-detection patterns in dual-target- and ≥3-target-positive specimens. **(A)** Exact dual-target co-detection combinations observed in at least five specimens in the main 4-year non-SARS-CoV-2 analysis; bars show specimen counts for each target pair. **(B)** Target occurrence frequencies within ≥3-target-positive specimens. **(C)** Most frequent exact ≥3-target combinations. Filled circles indicate detected targets and open circles indicate absent targets; less frequent combinations are grouped as “Other ≥3-target combinations”. SARS-CoV-2-containing dual combinations were excluded from Panel **(A)**. Because the ≥3-target-positive stratum was small (*n* = 56), Panels **(B, C)** are presented descriptively.

Respiratory viral co-detection patterns are summarized in Figure 4. Among 508 dual-target-positive specimens in the main analysis, 29 exact dual-target combinations had frequencies of at least five specimens and together accounted for 431 dual-positive specimens (84.8%). The most frequent exact dual-target combination was E-RV + AdV (*n* = 71; 14.0% of dual-positive specimens), followed by AdV + IAV (*n* = 31; 6.1%), E-RV + HCoV-OC43 (*n* = 29; 5.7%), E-RV + RSV A/B (*n* = 27; 5.3%), E-RV + HPIV-3 (*n* = 25; 4.9%), and AdV + RSV A/B (*n* = 23; 4.5%). The complete age-stratified distribution of exact dual-target co-detection combinations is provided in Supplementary Data Sheet 1 and Supplementary Table 5.

Among the 56 specimens with ≥3-target positivity, E-RV was the most frequent component, detected in 41/56 specimens (73.2%). Other frequent components were AdV (31/56), RSV A/B (23/56), HBoV (19/56), HCoV-OC43 (19/56), and IAV (18/56). The five most frequent exact ≥3-target combinations accounted for 18/56 specimens (32.1%), whereas the remaining 38/56 specimens (67.9%) consisted of heterogeneous lower-frequency combinations. These findings indicate that complex co-detection was not driven by a single dominant ≥3-target cluster. The complete distribution of exact ≥3-target co-detection combinations is provided in Supplementary Data Sheet 1 and Supplementary Table 6.

### 3.4 Age-stratified seasonal dynamics of selected viral targets

**Figure 5:**
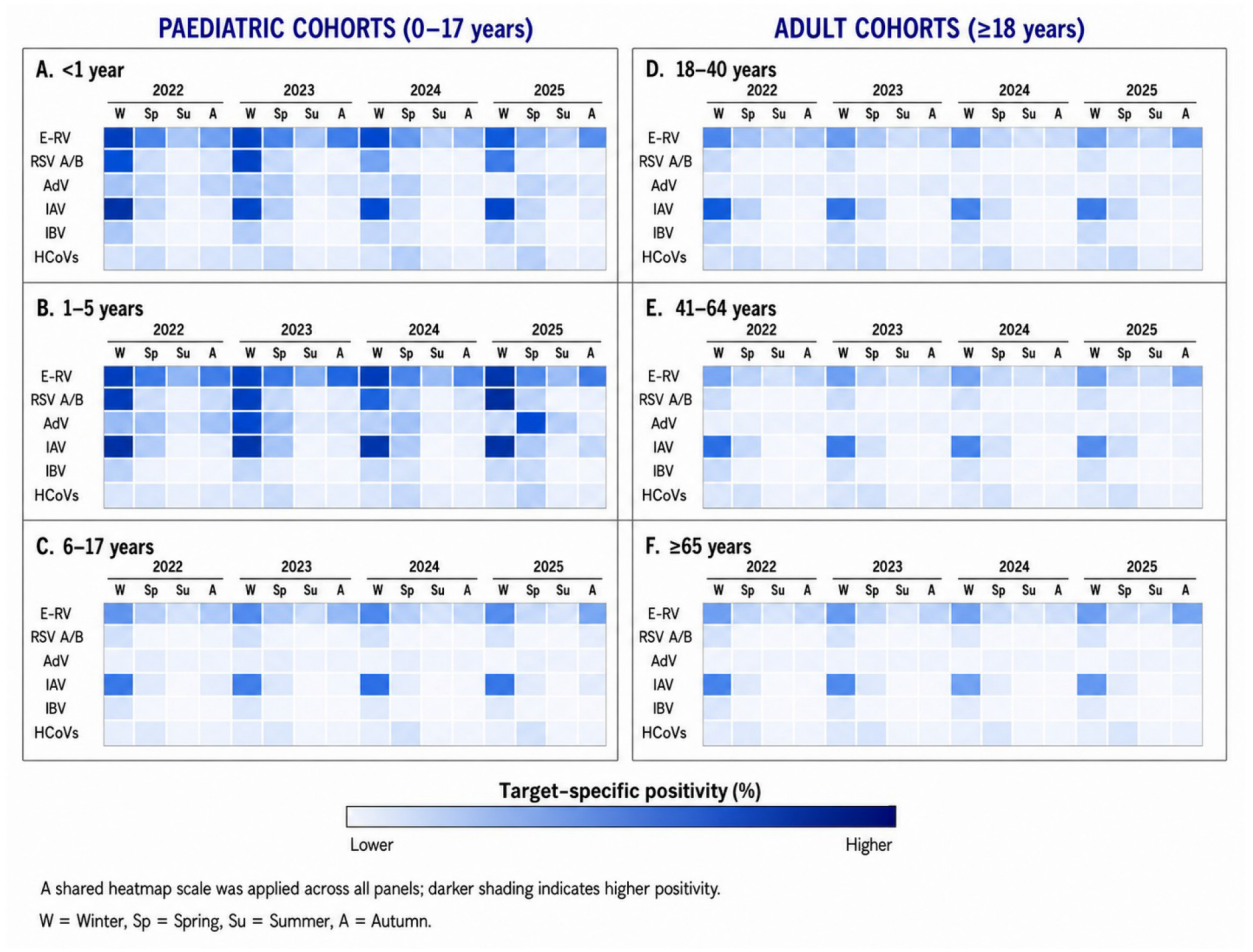
Age-stratified seasonal positivity patterns of selected non-SARS-CoV-2 respiratory viral targets, 2022–2025. Heatmaps show target-specific positivity rates among specimens tested within each age group, calendar year, and season in the main non-SARS-CoV-2 analysis. Panels **(A–C)** represent pediatric age groups and panels **(D–F)** represent adult age groups. Viral targets were restricted to those with complete testing coverage throughout 2022–2025 and at least 100 detections during the study period. A shared color scale is used across all panels; darker shading indicates higher target-specific positivity. SARS-CoV-2 findings, limited to the 2024–2025 multiplex-panel period, are presented separately in the Supplementary material. W, winter; Sp, spring; Su, summer; A, autumn.

Age-stratified seasonal dynamics for respiratory viral targets with full 2022–2025 testing coverage are shown in Figure 5. The selected targets showed distinct seasonal and age-related patterns.

E-RV had the broadest distribution, being detected across all age groups and seasons, with its highest aggregate seasonal positivity in autumn (658/6,264; 10.5%). IAV showed the most pronounced winter predominance, with winter positivity of 617/6,641 (9.3%) and only rare detection during summer (1/4,482; 0.02%). RSV A/B was also winter-weighted (376/6,641; 5.7%) and was most evident in younger children.

Age-related variation was also apparent across viral targets. AdV and E-RV were prominent in pediatric age groups, particularly among children aged 1–5 years, whereas IAV was more evident during winter in school-aged children and adult age groups. IBV showed a lower-intensity and more intermittent seasonal profile. Aggregated seasonal HCoV detections were more common in winter and spring than in summer.

SARS-CoV-2 restricted 2024–2025 seasonal sensitivity outputs are provided in Supplementary Data Sheet 1 and Supplementary Table 7. Detailed year-, season-, and age-stratified viral target detection frequencies and positivity-pattern distributions are provided in Supplementary Data Sheet 2 and Supplementary Tables 9, 10.

### 3.5 Meteorological exposure-window associations

**Figure 6:**
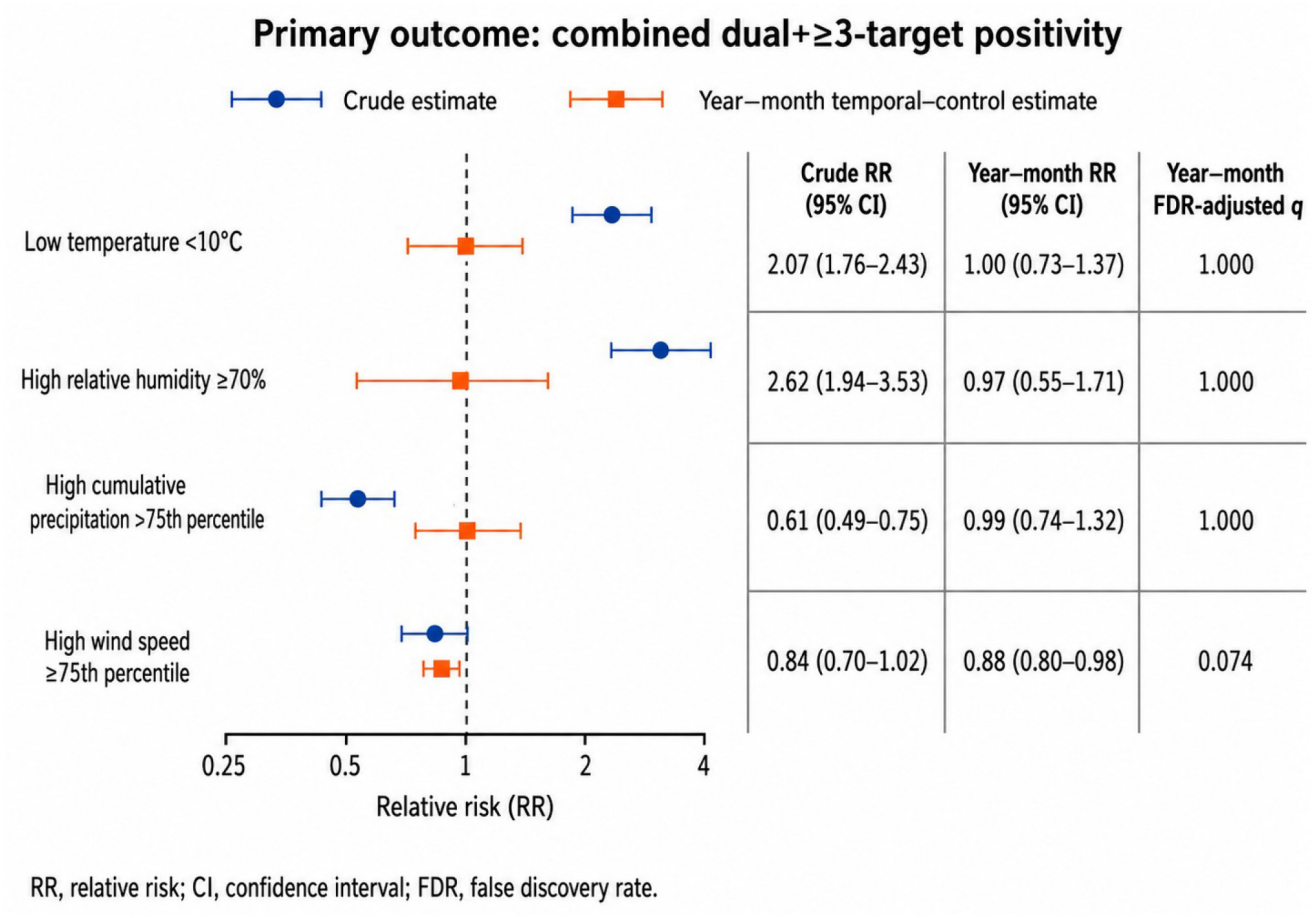
Meteorological associations with combined dual+≥3-target positivity. Crude and year–month temporal-control relative risk estimates are shown for combined dual+≥3-target positivity, the prespecified primary environmental outcome. Points and horizontal bars indicate relative risks and 95% confidence intervals for each meteorological exposure category; the dashed vertical line marks RR = 1. The accompanying table summarizes the corresponding crude and temporal-control estimates together with year–month FDR-adjusted q values. Temporal-control estimates assess whether crude associations were retained after restricting comparisons to the same calendar year–month strata.

Meteorological associations were evaluated within the predefined incubation-informed 1–9-day pre-sampling exposure window, using combined dual+≥3-target positivity as the primary environmental outcome (Figure 6).

In crude analyses, low temperature (<10 °C) and high relative humidity (≥70%) were associated with higher dual+≥3-target positivity, with RR estimates of 2.07 (95% CI, 1.76–2.43) and 2.62 (95% CI, 1.94–3.53), respectively. High cumulative precipitation (≥75th percentile) showed an inverse association (RR = 0.61; 95% CI, 0.49–0.75), whereas high wind speed (≥75th percentile) showed little crude association (RR = 0.84; 95% CI, 0.70–1.02).

After year–month temporal control, the low-temperature, high-humidity, and precipitation estimates were attenuated toward the null. High wind speed remained directionally inverse (RR = 0.88; 95% CI, 0.80–0.98; nominal *p* = 0.018; FDR-adjusted q = 0.074), but did not meet the FDR criterion.

These findings indicate that the crude meteorological signals were largely structured by shared seasonality and calendar time. A descriptive ecological sensitivity analysis of overall monthly non-SARS-CoV-2 viral positivity and monthly mean temperature is provided in Supplementary Data Sheet 3 and Supplementary Table 12, and was not interpreted as evidence of an independent environmental association. Complete crude and temporal-control outputs, including nominal *p*-values, FDR-adjusted q values, and exact-count outputs for the sparse ≥3-target category, are provided in Supplementary Data Sheet 3.

### 3.6 Pollutant-specific air-quality exposure-window associations

**Figure 7:**
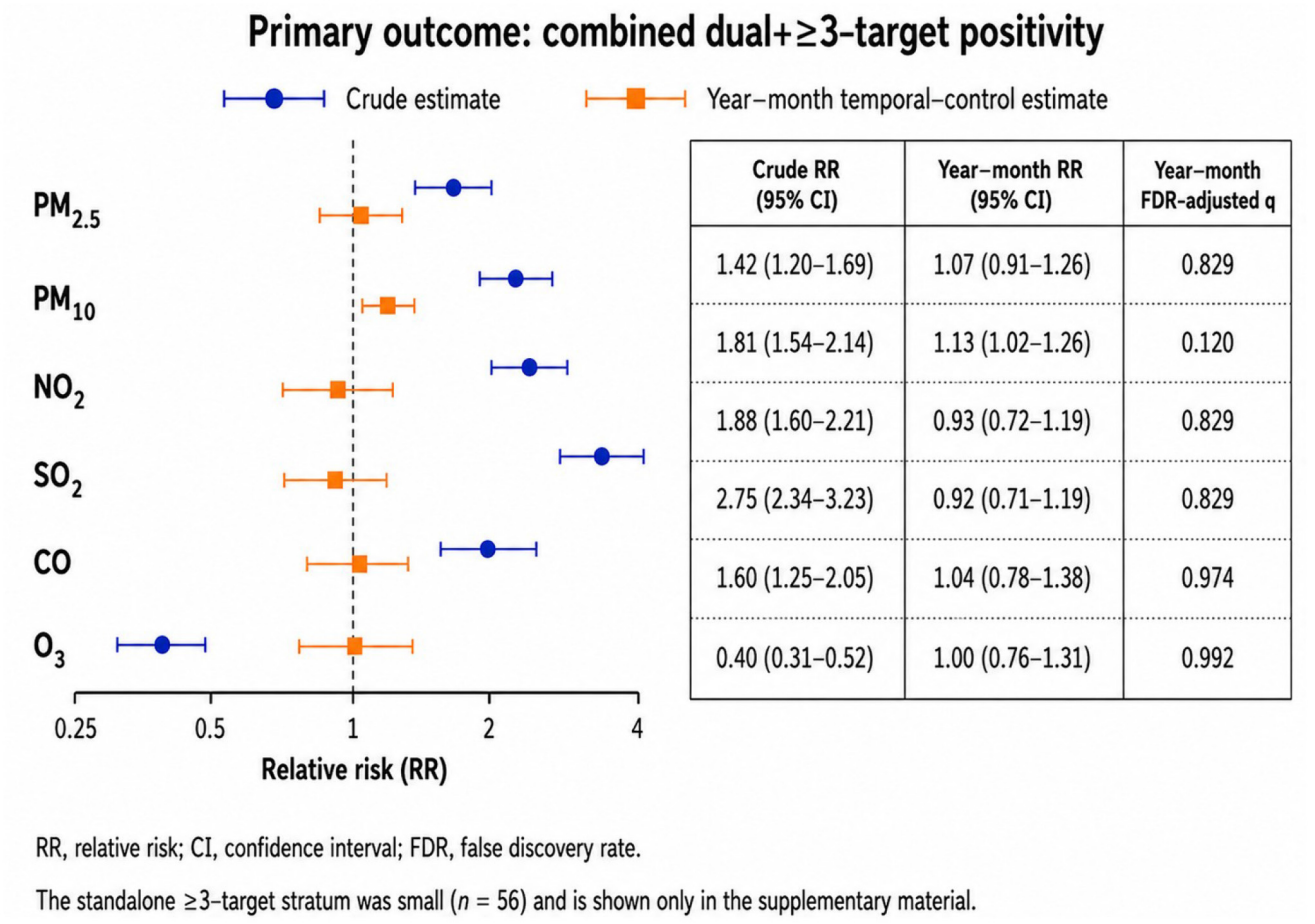
Pollutant-specific air-quality associations with combined dual+≥3-target positivity. Crude and year–month temporal-control relative risk estimates are shown for combined dual+≥3-target positivity, the prespecified primary environmental outcome. Analyses were based on pollutant-specific high-exposure categories within the predefined 1–9-day pre-sampling window. Points and horizontal bars indicate relative risks and 95% confidence intervals; the dashed vertical line marks RR = 1. The accompanying table summarizes the corresponding crude and temporal-control estimates together with year–month FDR-adjusted q values. Temporal-control estimates assess whether crude associations were retained after restricting comparisons to the same calendar year–month strata.

Pollutant-specific air-quality associations were evaluated within the same predefined 1–9-day pre-sampling exposure window, using combined dual+≥3-target positivity as the primary environmental outcome (Figure 7).

In crude analyses, high PM_2.5_, PM_10_, NO_2_, SO_2_, and CO exposures were associated with higher dual+≥3-target positivity, whereas high O_3_ exposure showed an inverse association. Figure 7 summarizes the crude and year–month temporal-control estimates. Complete pollutant-specific exposure-window results are provided in Supplementary Data Sheet 1 and Supplementary Table 8, with additional temporal-control and sparse ≥3-target outputs in Supplementary Data Sheet 3.

After year–month temporal control, all pollutant-specific estimates were attenuated, and none met the FDR criterion. PM10 remained directionally positive (RR = 1.13; 95% CI, 1.02–1.26; nominal *p* = 0.020; FDR-adjusted q = 0.120). For the remaining pollutants, temporal-control RRs ranged from 0.92 to 1.07, all confidence intervals included the null, and FDR-adjusted q values ranged from 0.829 to 0.992.

Taken together, the pollutant-specific findings were interpreted as exploratory contextual signals rather than confirmatory evidence of independent short-term environmental effects.

## 4 Discussion

### 4.1 Principal findings and comparative context

This study evaluated respiratory viral detections in 21,151 clinician-requested respiratory specimens tested at a tertiary-care hospital in Istanbul during 2022–2025. In the main analysis excluding SARS-CoV-2, 4,836 specimens (22.9%) were positive for at least one evaluated respiratory viral target. Accordingly, the findings describe molecular detection patterns in a clinically tested population rather than community-level respiratory virus incidence.

The dominant patterns were related to age, viral target, and season, whereas overall viral positivity did not differ meaningfully by sex. Non-SARS-CoV-2 target-positive specimens were obtained from younger patients than target-negative specimens, and children aged 1–5 years contributed disproportionately to single-target, dual-target, and ≥3-target positivity. Enterovirus/rhinovirus was the most frequently detected target and was represented across both single-target and co-detection patterns, underscoring its broad contribution to the molecular detection profile.

These findings support an age- and target-stratified interpretation of multiplex respiratory viral testing data rather than reliance on aggregate positivity alone. In comparison with recent multiplex respiratory panel studies, the present cohort showed a non-SARS-CoV-2 viral detection rate within the broad range reported in clinically tested populations, although direct numerical comparison is limited by differences in study period, age structure, testing population, panel composition, SARS-CoV-2 handling, geography, and seasonal timing (Table 1). This is particularly relevant for studies spanning the pandemic and post-pandemic periods, when diagnostic workflows, healthcare-seeking behavior, public health measures, and SARS-CoV-2 circulation may have influenced the detection of other respiratory viruses.

**Table 1:** Selected multiplex respiratory panel studies and reported detection measures according to SARS-CoV-2 handling.

Study context	Platform	*N*	SARS-CoV-2 situation	Reported detection measure	Predominant targets reported
Smedberg et al., 2022; USA	BioFire Respiratory Panel 2.0	1,783	SARS-CoV-2 negative cohort during the Alpha-surge period	At least one respiratory virus detected in 373/1783 specimens (20.9 %)	E-RV; seasonal HCoVs
Duclos et al., 2022; international	BioFire Respiratory Panel 2.0 Plus	1,334	SARS-CoV-2 negative cohort by study design	Viral or bacterial target detected in 484/1334 patients (36.3%)	E-RV; seasonal HCoVs; AdV
Li et al., 2023; China	Designed multiplex probe amplification; limited NGS validation	1,070	SARS-CoV-2 included in assay	At least one respiratory virus detected in 95/1070 samples (9%)	SARS-CoV-2; RSV; HRV / IAV
Karabulut et al., 2024; Türkiye	Bio-Speedy RT-PCR MX-24S	11,048	SARS-CoV-2 included in assay/reporting framework during pandemic/post-pandemic period	Viral-only category among pathogen-positive specimens: 3,779/7,807 (48.4%)	E-RV; RSV A/B; SARS-CoV-2; IAV
Koçer et al., 2025; Türkiye	QIAstat-Dx Respiratory Panel	748	SARS-CoV-2 included in assay; pandemic/post-pandemic periods analyzed	Overall viral positivity reported 326/748 specimens (43.6%)	E-RV; RSV; SARS-CoV-2
Onel et al., 2025; Türkiye	QIAstat-Dx Respiratory Panel	1,984	SARS-CoV-2 included in assay/reporting framework during pandemic/post-pandemic period	Single viral agent detected in 959/1,984 patients (48.3%)	E-RV; SARS-CoV-2; influenza A
Present study	Bio-Speedy RT-PCR MX-24S	21,151	SARS-CoV-2 excluded from main analyses	4,836/21,151 specimens (22.9%)	E-RV; AdV; RSV A/B

N denotes the total number of respiratory specimens or patients included in each study, as reported by the original authors. Values are presented according to each article's reporting framework and were not recalculated into a uniform metric. Because study populations, specimen units, panel composition, testing periods, SARS-CoV-2 inclusion/exclusion, and denominator definitions differed across studies, the summarized measures should be interpreted descriptively rather than as directly comparable estimates of non-SARS-CoV-2 viral positivity. In SARS-CoV-2-negative cohorts, SARS-CoV-2 testing was performed as part of the study selection process or outside the reported multiplex respiratory panel outcome; therefore, reported yields reflect respiratory panel findings in SARS-CoV-2-negative specimens/patients.

AdV, adenovirus; E-RV, enterovirus/rhinovirus; HCoVs, human coronaviruses; HRV/EV or RV/EV, human rhinovirus/enterovirus or rhinovirus/enterovirus; IAV, influenza A virus; NGS, next-generation sequencing; RP, respiratory panel; RSV, respiratory syncytial virus.

The environmental analyses provided contextual information about detection complexity but did not supersede the dominant age-, target-, and season-related patterns. Crude associations between short-term meteorological or pollutant-specific exposures and combined dual+≥3-target positivity were substantially attenuated after year–month temporal control, supporting their interpretation as temporally structured contextual signals rather than independent short-term effects.

### 4.2 Age-stratified detection and molecular co-detection patterns

A major age-stratified finding of this cohort was the concentration of molecular detection complexity in early childhood. Children aged 1–5 years contributed the largest shares of single-target, dual-target, and ≥3-target detections, indicating that both overall positivity and multi-target detection were strongly age patterned. This distribution is consistent with pediatric respiratory virus studies reporting higher detection frequencies in young children, probably reflecting close-contact environments, household and daycare or school-based exposure, and limited accumulated immune experience (Smits and Jochems, 2024; Chen et al., 2020; Yaman et al., 2022).

At the same time, the balance between single-target positivity and co-detection differs substantially across multiplex respiratory panel studies (Table 1). Reported single-target proportions among positive specimens range from below 50% in some Türkiye-based cohorts to approximately 90% or higher in other multiplex respiratory panel studies. The present cohort showed a predominantly single-target profile, with 88.3% single-target positivity among non-SARS-CoV-2 target-positive specimens. Such variation is unlikely to reflect differences in viral biology alone. It may also reflect differences in age structure, clinical setting, testing indication, panel composition, sampling period, SARS-CoV-2 handling, and whether repeated or persistent PCR detections were excluded. Therefore, the high contribution of the 1–5-year group and the predominance of single-target positivity in the present cohort should be interpreted as characteristics of an age-structured, clinician-requested tertiary-care testing dataset rather than as direct estimates of community co-infection burden. Because the standalone ≥3-target category comprised only 56 specimens, it was interpreted descriptively.

Throughout this study, detection of more than one viral target was interpreted as molecular co-detection rather than confirmed clinical co-infection. Multiplex RT-PCR identifies viral nucleic acid in the same specimen but cannot determine viral viability, symptom causality, duration of shedding, or direct biological interaction between detected viral targets (Piret and Boivin, 2022).

Adenovirus plus enterovirus/rhinovirus was the most frequent dual-target combination. This pairing occurred within the broader pediatric pattern of frequent adenovirus and enterovirus/rhinovirus detection, and may therefore reflect the overlap of common childhood viral exposures within the tested clinical population. Its interpretation nevertheless requires caution because adenoviral nucleic acid may persist in the upper respiratory tract, and repeated PCR positivity may reflect either new detection or prolonged nucleic-acid persistence rather than concurrent clinically active infection (Kalu et al., 2010).

Enterovirus/rhinovirus was represented across single-target, dual-target, and ≥3-target structures, in keeping with its broad age distribution and wider seasonal presence relative to more sharply seasonal respiratory viruses (Piret and Boivin, 2022; Pierangeli et al., 2025). In contrast, exact ≥3-target combinations were heterogeneous, and no single pattern accounted for a substantial proportion of this sparse category. Taken together, these findings suggest that molecular co-detection in this cohort was shaped by overlapping pediatric susceptibility, concurrent seasonal circulation, and clinician-requested testing structure rather than by one dominant target combination.

Human metapneumovirus showed a relatively high proportional representation within dual+≥3-target detections, despite a limited absolute number of detections. This should be interpreted as a descriptive feature of the multi-target detection structure rather than evidence that hMPV was a major driver of overall detection complexity. The pediatric and seasonal pattern observed in the present cohort is broadly consistent with recent post-pandemic surveillance and pediatric clinical data. In a 4-year single-center pediatric study from Türkiye, Öcal Demir et al. reported 78 hMPV-related acute respiratory infection cases, of which 84.6% occurred in children younger than 5 years, 92.3% presented during winter or spring, and 29.5% had viral co-infection (Öcal Demir et al., 2026). These findings support the interpretation that hMPV detections are concentrated in young children and cooler-season periods, although its apparent contribution to multi-target detection may vary across studies according to healthcare setting, testing indication, age composition, panel design, and whether analyses include all multiplex-positive specimens or only hMPV-positive pediatric cases.

### 4.3 Seasonal dynamics and SARS-CoV-2 restriction

Seasonal detection patterns differed substantially by viral target and age group. Among targets assessed consistently throughout 2022–2025, influenza A virus showed the clearest winter predominance, RSV A/B followed a cooler-season and pediatric-weighted profile, whereas enterovirus/rhinovirus was detected across a wider range of seasons and age strata. This contrast indicates that aggregate positivity estimates can mask biologically and epidemiologically distinct target-specific patterns, including seasonal peaks, age distributions, and co-detection structures. The broad distribution of enterovirus/rhinovirus, together with the winter-weighted profiles of influenza A virus and RSV A/B, is consistent with the recognized heterogeneity of respiratory virus seasonality in temperate settings (Bloom-Feshbach et al., 2013; Neumann and Kawaoka, 2022).

SARS-CoV-2-specific analyses were restricted to the 2024–2025 multiplex-panel period because comparable multiplex-panel data were not available across the full 4-year study period. Within this restricted window, SARS-CoV-2 showed a summer–autumn-weighted distribution and was detected predominantly as a single target. This finding should be interpreted as a period-specific sensitivity observation rather than directly compared with viral targets assessed consistently throughout 2022–2025. Differences in testing availability, variant dynamics, population immunity, healthcare-seeking behavior, and calendar-time structure may all have influenced the observed pattern.

The seasonal behavior of ozone further illustrates the importance of interpreting environmental signals within the underlying viral seasonality. O_3_ concentrations were higher during warmer and sunnier periods, whereas influenza A virus and RSV A/B were concentrated mainly in colder seasons in this cohort. The inverse crude association between high O_3_ exposure and combined dual+≥3-target positivity was no longer evident after year–month temporal control, supporting its interpretation as a seasonally structured ecological signal rather than evidence of a protective biological effect.

### 4.4 Meteorological exposure-window findings

Low temperature and high relative humidity were associated with higher dual+≥3-target positivity in crude analyses, whereas high cumulative precipitation showed an inverse association. These estimates were substantially attenuated after year–month temporal control, indicating that the crude patterns were largely structured by shared seasonality and calendar time. High wind speed remained directionally inverse after temporal control, but did not meet the FDR-adjusted criterion (q = 0.074).

The predefined 1–9-day pre-sampling window provided a biologically informed framework for examining short-term environmental conditions in relation to molecular detection of respiratory viral targets. However, because exposures were anchored to specimen collection rather than symptom onset, the estimates cannot be interpreted as pathogen-specific incubation-period effects. In this clinician-requested testing cohort, the crude low-temperature signal is therefore best regarded as a marker of seasonal conditions accompanying higher combined dual+≥3-target positivity, potentially together with indoor contact, school attendance, age composition, testing behavior, and other unmeasured factors.

The descriptive monthly analysis of overall non-SARS-CoV-2 viral positivity and mean temperature was likewise not interpreted as evidence of an independent short-term environmental association because both measures shared substantial seasonal and calendar-time structure. Given the absence of symptom-onset dates, clinician-requested rather than standardized testing, and variation in incubation profiles and event counts across viral targets, distributed lag non-linear models were not considered appropriate for the present dataset. Prospective studies with onset-dated data, standardized testing indications, and adequate target-specific event numbers would be better suited to distributed-lag modeling (Gasparrini et al., 2010). Within the present specimen-based dataset, the 1–9-day window was therefore retained as a pragmatic exploratory exposure framework (Spencer et al., 2022; Gressani et al., 2025; Tsinaris et al., 2026).

### 4.5 Pollutant-specific air-quality findings

High PM_2.5_, PM_10_, NO_2_, SO_2_, and CO exposures showed positive crude associations with combined dual+≥3-target positivity, whereas high O_3_ exposure showed an inverse crude association. After year–month temporal control, most pollutant-specific estimates were attenuated toward the null, and none met the FDR criterion. PM_10_ remained directionally positive, but without FDR-adjusted evidence (RR = 1.13; 95% CI, 1.02–1.26; q = 0.120).

These findings are compatible with previous evidence linking air pollution to respiratory infection susceptibility and respiratory infection-related healthcare use, but they support a cautious interpretation in the present dataset. The pollutants evaluated in this study overlap with the major air-quality indicators highlighted in the WHO global air quality guidelines, including PM_2.5_, PM_10_, O_3_, NO_2_, SO_2_, and CO (World Health Organization, 2021). In pediatric settings, Choi et al. reported that seasonal co-exposure to air pollutants and allergenic pollens may enhance susceptibility to respiratory viral infections in children (Choi et al., 2022). More broadly, pollutant-related effects on airway inflammation, oxidative stress, mucosal defense, and host antiviral responses provide biologically plausible pathways linking air pollution with respiratory infections, particularly in children (Esposito et al., 2025).

However, the present specimen-level data cannot separate these potential mechanisms from seasonal co-variation, pollutant co-variation, testing behavior, indoor exposure, mobility, or other unmeasured contextual factors. In addition, prior studies have often evaluated clinical respiratory outcomes, hospital admissions, or respiratory viral infection counts rather than multiplex PCR-confirmed molecular co-detection patterns. Accordingly, the pollutant-specific findings in the present study should be interpreted as exploratory contextual associations rather than evidence of independent causal effects.

### 4.6 Statistical interpretation and outcome framework

Environmental findings were interpreted using effect size, confidence-interval precision, FDR-adjusted evidence, and year–month temporal-control results together, rather than nominal *p*-values alone. This was important because both environmental exposures and respiratory viral detection patterns showed strong seasonal and calendar-time structure. Benjamini–Hochberg correction was applied separately to crude and year–month temporal-control estimates within the prespecified meteorological and pollutant-specific exposure families, reducing the risk of overinterpreting isolated nominal associations while preserving the distinction between exposure domains (Benjamini and Hochberg, 1995; Tsinaris et al., 2026).

Combined dual+≥3-target positivity was prespecified as the primary environmental outcome because it summarized molecular detection complexity without relying on the sparse standalone ≥3-target stratum. This choice reduced principal comparisons, avoided treating nested positivity categories as independent primary endpoints, and aligned the environmental analysis with the specimen-level descriptive framework of the study. Other positivity categories were retained as secondary or descriptive outputs. Accordingly, these analyses should be interpreted as evaluating short-term exposure associations with molecular detection complexity, rather than estimating virus-specific environmental effects or pathogen–pathogen interactions.

Virus-specific meteorological models were not performed because they would have increased multiplicity, relied on uneven and sometimes sparse target-specific event counts, and been difficult to interpret without symptom-onset dates. Respiratory viruses also differ in incubation period, shedding duration, seasonal timing, and clinical testing patterns; therefore, short-window exposure models anchored to specimen collection date could have produced unstable or selectively interpretable target-specific findings. For this reason, the revised environmental framework was restricted to the prespecified combined dual+≥3-target outcome, while target-specific variation was described through age- and season-stratified analyses.

### 4.7 Strengths and limitations

This study has several strengths. It analyzed a large real-world molecular diagnostic dataset comprising 21,151 respiratory specimens from Istanbul, a densely populated metropolitan setting with substantial human mobility and marked seasonal variation. The multiplex RT-PCR platform enabled simultaneous assessment of multiple respiratory viral targets and allowed single-target, dual-target, and ≥3-target detection patterns to be evaluated across age groups, seasons, and calendar years.

A further strength was the integration of demographic, virological, seasonal, and environmental dimensions within the same clinical testing framework. This approach allowed respiratory viral detection to be examined beyond overall positivity rates and supported a more structured interpretation of age-stratified detection, molecular co-detection, and environmental context. Separating viral targets with full 2022–2025 testing coverage from restricted SARS-CoV-2-specific multiplex-panel outputs improved the interpretability of longitudinal comparisons. In addition, the predefined incubation-informed exposure window, FDR correction, and year–month temporal-control analyses strengthened the transparency and caution of the environmental interpretation.

Several limitations should also be considered. This was a single-center retrospective study based on clinician-requested testing; therefore, the findings describe patterns among tested specimens and should not be interpreted as community-level respiratory virus incidence. Patient-level clinical information was unavailable, including symptom onset, symptom duration, disease severity, comorbidities, immunosuppression, vaccination status, treatment, and clinical outcomes. As a result, the clinical relevance of individual viral targets and molecular co-detection patterns could not be determined.

Environmental exposure assignment was ecological rather than individual level. Meteorological and air-quality indicators were linked to specimen collection dates, not to personal exposure histories, indoor environments, household or school contacts, mobility patterns, or time spent in specific microenvironments. Because symptom-onset dates were unavailable, exposure windows were anchored to specimen collection rather than to illness onset. Detection complexity was also strongly age structured, so residual confounding by age composition within calendar periods cannot be fully excluded. In addition, CO had lower daily completeness than the other pollutants, which reduced the number of specimens with a valid CO exposure-window summary. Accordingly, CO-specific estimates should be interpreted with additional caution. Taken together, these factors limit causal interpretation and support viewing the environmental findings as contextual exposure associations.

Finally, the standalone ≥3-target category included only 56 specimens and was therefore interpreted descriptively. Comparable SARS-CoV-2-specific multiplex-panel data were unavailable across the full 2022–2025 period because earlier testing relied on different workflows and was not uniformly captured in the Laboratory Information Management System. This limited direct longitudinal comparison between SARS-CoV-2 and viral targets assessed consistently throughout the study period.

## 5 Conclusion

In this large clinician-requested multiplex RT-PCR dataset from Istanbul, respiratory viral detection patterns were shaped primarily by age, viral target, and season. Children aged 1–5 years contributed disproportionately to molecular detection complexity, whereas enterovirus/rhinovirus showed the broadest distribution across age groups and seasons. These findings highlight the value of moving beyond aggregate positivity rates by considering age-stratified patterns, molecular co-detection structures, and target-specific seasonality.

Exploratory analyses of short-term environmental exposures identified crude associations within the predefined 1–9-day pre-sampling window; however, most meteorological and pollutant-specific estimates were attenuated after year–month temporal control. Accordingly, the environmental findings should be interpreted as contextual signals shaped by seasonal and calendar-time structure rather than as evidence of independent causal effects.

Overall, routinely generated multiplex molecular data can provide a useful framework for interpreting respiratory viral detection patterns in large metropolitan clinical testing settings, particularly when demographic, seasonal, molecular, and environmental dimensions are considered together. Environmental associations should be considered exploratory unless supported by prospective, onset-dated, and exposure-resolved study designs.

## Data Availability

The original contributions presented in the study are included in the article/Supplementary material, further inquiries can be directed to the corresponding author.

## References

[B1] Al-Rousan N. Al-Najjar H. Elhaty I. A. (2025). Machine learning framework for forecasting air pollution: evaluating seasonal and climatic influences in Istanbul, Turkey. PLoS ONE 20:e0330716. doi: 10.1371/journal.pone.0330716 41082547 PMC12517522

[B2] Benjamini Y. Hochberg Y. (1995). Controlling the false discovery rate: a practical and powerful approach to multiple testing. J. R. Stat. Soc. Series B Stat. Methodol. 57, 289–300. doi: 10.1111/j.2517-6161.1995.tb02031.x

[B3] Bloom-Feshbach K. Alonso W. J. Charu V. Tamerius J. Simonsen L. Miller M. A. . (2013). Latitudinal variations in seasonal activity of influenza and respiratory syncytial virus (RSV): a global comparative review. PLoS ONE 8:e54445. doi: 10.1371/journal.pone.0054445 23457451 PMC3573019

[B4] Bostan P. Yavuz C. I. Öztürk B. Olcay S. S. Aykaç N. (2023). Correlation between daily PM10, nitrogen dioxide, and ozone measurements with the stringency index in 15 different districts of a big metropolis. Thorac. Res. Pract. 24, 253–261. doi: 10.5152/ThoracResPract.2023.22231 37581375 PMC10542101

[B5] Burbank A. J. (2023). Risk factors for respiratory viral infections: a spotlight on climate change and air pollution. J. Asthma Allergy 16, 183–194. doi: 10.2147/JAA.S364845 36721739 PMC9884560

[B6] Celik E. Gul M. (2022). How Covid-19 pandemic and partial lockdown decisions affect air quality of a city? The case of Istanbul, Turkey. Environ. Dev. Sustain. 24, 1616–1654. doi: 10.1007/s10668-021-01328-w 33776552 PMC7988252

[B7] Channappanavar R. Perlman S. (2020). Age-related susceptibility to coronavirus infections: role of impaired and dysregulated host immunity. J. Clin. Invest. 130, 6204–6213. doi: 10.1172/JCI144115 33085654 PMC7685716

[B8] Chen C. Liu H. M. Liu J. Y. Chen P. F. Lu H. Z. (2025). Changing epidemiology of respiratory pathogens since 2020: Shenzhen case study and global perspectives. Drug Discov. Ther. 19, 277–284. doi: 10.5582/ddt.2025.01108 41125353

[B9] Chen J. Kelley W. J. Goldstein D. R. (2020). Role of aging and the immune response to respiratory viral infections: potential implications for COVID-19. J. Immunol. 205, 313–320. doi: 10.4049/jimmunol.2000380 32493812 PMC7343582

[B10] Choi Y.-J. Lee K. S. Lee Y.-S. Kim K. R. Oh J.-W. (2022). Analysis of the association among air pollutants, allergenic pollen, and respiratory virus infection of children in Guri, Korea during recent 5 years. Allergy Asthma Immunol. Res. 14, 289–299. doi: 10.4168/aair.2022.14.3.289 35557494 PMC9110915

[B11] Ciencewicki J. Jaspers I. (2007). Air pollution and respiratory viral infection. Inhal. Toxicol. 19, 1135–1146. doi: 10.1080/08958370701665434 17987465

[B12] Couto P. Campbell H. Li Y. Rondy M. Leite J. Rodriguez A. . (2025). Characterisation of the respiratory syncytial virus seasonality and its environmental factors in the Americas: a multi-country observational study using routine surveillance networks. Lancet Reg. Health Am. 48:101166. doi: 10.1016/j.lana.2025.101166 40688765 PMC12273578

[B13] Dönmez I. Aslan Z. (2023). Role of air pollution on COVID-19 in Istanbul. Sigma J. Eng. Nat. Sci. 41, 793–806. doi: 10.14744/sigma.2022.00021

[B14] Duclos M. Hommel B. Allantaz F. Powell M. Posteraro B. Sanguinetti M. (2022). Multiplex PCR detection of respiratory tract infections in SARS-CoV-2-negative patients admitted to the emergency department: an international multicenter study during the COVID-19 pandemic. Microbiol. Spectr. 10:e02368-22. doi: 10.1128/spectrum.02368-22 36154273 PMC9603986

[B15] Esposito S. Fainardi V. Titolo A. Lazzara A. Menzella M. Campana B. . (2025). How air pollution fuels respiratory infections in children: current insights. Front. Public Health 13:1567206. doi: 10.3389/fpubh.2025.1567206 40365435 PMC12070440

[B16] Gasparrini A. Armstrong B. Kenward M. G. (2010). Distributed lag non-linear models. Stat. Med. 29, 2224–2234. doi: 10.1002/sim.3940 20812303 PMC2998707

[B17] Gressani O. Torneri A. Hens N. Faes C. (2025). Flexible Bayesian estimation of incubation times. Am. J. Epidemiol. 194, 490–501. doi: 10.1093/aje/kwae192 38988237 PMC11815507

[B18] Guarnieri G. Olivieri B. Senna G. Vianello A. (2023). Relative humidity and its impact on the immune system and infections. Int. J. Mol. Sci. 24:9456. doi: 10.3390/ijms24119456 37298409 PMC10253274

[B19] He Y. Liu W. J. Jia N. Richardson S. Huang C. (2023). Viral respiratory infections in a rapidly changing climate: the need to prepare for the next pandemic. EBioMedicine 93:104593. doi: 10.1016/j.ebiom.2023.104593 37169688 PMC10363434

[B20] Hersbach H. Bell B. Berrisford P. Hirahara S. Horányi A. Muñoz-Sabater J. . (2020). The ERA5 global reanalysis. Q. J. R. Meteorol. Soc. 146, 1999–2049. doi: 10.1002/qj.3803

[B21] Huang D. Taha M. S. Nocera A. L. Workman A. D. Mueller S. K. Bleier B. S. . (2023). Cold exposure impairs extracellular vesicle swarm-mediated nasal antiviral immunity. J. Allergy Clin. Immunol. 151, 509–525.e8. doi: 10.1016/j.jaci.2022.09.037 36494212 PMC13380700

[B22] Kalu S. U. Loeffelholz M. Beck E. Patel J. A. Revai K. Fan J. . (2010). Persistence of adenovirus nucleic acids in nasopharyngeal secretions: a diagnostic conundrum. Pediatr. Infect. Dis. J. 29, 746–750. doi: 10.1097/INF.0b013e3181d743c8 20308936 PMC3206289

[B23] Karabulut N. Alaçam S. Sen E. Karabey M. Yakut N. (2024). The epidemiological features and pathogen spectrum of respiratory tract infections, Istanbul, Türkiye, from 2021 to 2023. Diagnostics 14:1071. doi: 10.3390/diagnostics14111071 38893598 PMC11171886

[B24] Koçer I. Demirbakan H. Aktaş A. (2025). Temporal dynamics and forecasting of respiratory viral infections during and after the SARS-CoV-2 pandemic (2020–2027): a multiplex PCR and ARIMA-based study. Front. Microbiol. 16:1674529. doi: 10.3389/fmicb.2025.1674529 41078527 PMC12507870

[B25] Krivonosov M. I. Pazukhina E. Zaikin A. Viozzi F. Lazzareschi I. Manca L. . (2024). Evaluating the effect of climate on viral respiratory diseases among children using AI. J. Clin. Med. 13:7474. doi: 10.3390/jcm13237474 39685931 PMC11641916

[B26] Li C. Xi L. Rao J. Tang F. Xiang Y. Wang X. (2025). Epidemiological trends and climatic drivers of pediatric respiratory infections in Wuhan, China: a multi-pathogen analysis. Front. Cell. Infect. Microbiol. 15:1624638. doi: 10.3389/fcimb.2025.1624638 40980014 PMC12443746

[B27] Li M. Li Q. Yang G. Kolosov V. P. Perelman J. M. Zhou X. D. (2011). Cold temperature induces mucin hypersecretion from normal human bronchial epithelial cells in vitro through a TRPM8-mediated mechanism. J. Allergy Clin. Immunol. 128, 626–634.e5. doi: 10.1016/j.jaci.2011.04.032 21762971

[B28] Li W. Wang X. Cui W. Yuan L. Hu X. (2023). Clinical evaluation of a multiplex PCR assay for simultaneous detection of 18 respiratory pathogens in patients with acute respiratory infections. Pathogens 12:21. doi: 10.3390/pathogens12010021 36678368 PMC9862116

[B29] Lowen A. C. Mubareka S. Steel J. Palese P. (2007). Influenza virus transmission is dependent on relative humidity and temperature. PLoS Pathog. 3:e151. doi: 10.1371/journal.ppat.0030151 17953482 PMC2034399

[B30] Matera L. Manti S. Petrarca L. Pierangeli A. Conti M. G. Mancino E. . (2023). An overview on viral interference during the SARS-CoV-2 pandemic. Front. Pediatr. 11:1308105. doi: 10.3389/fped.2023.1308105 38178911 PMC10764478

[B31] Menteşe S. Ogurtani S. O. (2022). Spatial and temporal look at ten-years air quality of Istanbul city. Int. J. Environ. Sci. Technol. 19, 925–938. doi: 10.1007/s13762-020-03061-9

[B32] Mese S. Allahverdiyeva A. Onel M. Uysal H. K. Agacfidan A. (2024). Investigation of the effect of the COVID-19 pandemic period on respiratory tract viruses at Istanbul Medical Faculty Hospital, Turkey. Infect. Dis. Rep. 16, 992–1004. doi: 10.3390/idr16050079 39452164 PMC11507061

[B33] Neumann G. Kawaoka Y. (2022). Seasonality of influenza and other respiratory viruses. EMBO Mol. Med. 14:e15352. doi: 10.15252/emmm.202115352 35157360 PMC8988196

[B34] Öcal Demir S. Yilmaz S. Dizi Işik A. Çagla Abaci Çapar M. Cura S. Dedeoglu R. . (2026). Characteristics of human metapneumovirus-related acute respiratory infections in children: a four-year single center retrospective study. Jpn. J. Infect. Dis. doi: 10.7883/yoken.JJID.2026.015. [Epub ahead of print]. 42055699

[B35] Onel M. Uysal H. K. Hulikyan A. Ucar Y. A. Yapar G. Allahverdiyeva A. . (2025). Syndromic testing in the pandemic era and beyond: rapid detection for respiratory infections in Istanbul. Viruses 17:776. doi: 10.3390/v17060776 40573366 PMC12197672

[B36] Orak N. H. (2022). Effect of ambient air pollution and meteorological factors on the potential transmission of COVID-19 in Turkey. Environ. Res. 212:113646. doi: 10.1016/j.envres.2022.113646 35688216 PMC9172252

[B37] Pierangeli A. Turriziani O. Fracella M. Campagna R. Frasca F. D'Auria A. . (2025). The added value of diagnostics to characterize age-specific patterns of respiratory viral infections and coinfections and to detect emerging threats. BMC Infect. Dis. 25:404. doi: 10.1186/s12879-025-10693-0 40133829 PMC11934565

[B38] Piret J. Boivin G. (2022). Viral interference between respiratory viruses. Emerg. Infect. Dis. 28, 273–281. doi: 10.3201/eid2802.211727 35075991 PMC8798701

[B39] Poletti P. Tirani M. Cereda D. Trentini F. Guzzetta G. Sabatino G. . (2021). Association of age with likelihood of developing symptoms and critical disease among close contacts exposed to patients with confirmed SARS-CoV-2 infection in Italy. JAMA Netw. Open 4:e211085. doi: 10.1001/jamanetworkopen.2021.1085 33688964 PMC7948061

[B40] Poniedziałek B. Rzymski P. Zarebska-Michaluk D. Flisiak R. (2024). Viral respiratory infections and air pollution: a review. Chemosphere 364:143096. doi: 10.1016/j.chemosphere.2024.142256 38723686

[B41] Price R. H. M. Graham C. Ramalingam S. (2019). Association between viral seasonality and meteorological factors. Sci. Rep. 9:929. doi: 10.1038/s41598-018-37481-y 30700747 PMC6353886

[B42] Republic of Türkiye Ministry of Environment Urbanization and Climate Change. (2026). National Air Quality Monitoring Network. Available online at: https://www.turkiye.gov.tr/cevre-ve-sehircilik-ulusal-hava-kalite-izleme-agi (accessed May 21, 2026).

[B43] Saadi S. Kallala O. Fodha I. Jerbi A. BenHamida-Rebai M. Hadj Fredj M. B. . (2021). Correlation between children respiratory virus infections and climate factors. J. Pediatr. Infect. Dis. 16, 67–73. doi: 10.1055/s-0040-1722569

[B44] Shi S. Lin H. Jiang L. Zeng Z. Lin C. Li P. . (2025). Development of a respiratory virus risk model with environmental data based on interpretable machine learning methods. npj Clim. Atmos. Sci. 8:39. doi: 10.1038/s41612-025-00894-4

[B45] Smedberg J. R. DiBiase L. M. Hawken S. E. Allen A. Mohan S. Santos C. . (2022). Reduction and persistence of co-circulating respiratory viruses during the SARS-CoV-2 pandemic. Am. J. Infect. Control 50, 1064–1066. doi: 10.1016/j.ajic.2022.06.008 35709970 PMC9188982

[B46] Smits H. H. Jochems S. P. (2024). Diverging patterns in innate immunity against respiratory viruses during a lifetime: lessons from the young and the old. Eur. Respir. Rev. 33:230266. doi: 10.1183/16000617.0266-2023 39009407 PMC11262623

[B47] Spencer J. A. Shutt D. P. Moser S. K. Clegg H. Wearing H. J. Mukundan H. . (2022). Distinguishing viruses responsible for influenza-like illness. J. Theor. Biol. 545:111145. doi: 10.1016/j.jtbi.2022.111145 35490763

[B48] Tsinaris Z. Meletis G. Tychala A. Theocharidou C. C. Greco A. Chatziantoniou T. . (2026). Decoding weather fingerprints of upper respiratory pathogens: a one-year observational study in Northern Greece. Curr. Microbiol. 83:6. doi: 10.1007/s00284-025-04589-0 41205066

[B49] World Air Quality Index Project (2026). World Air Quality Index Project. Available online at: https://waqi.info/ (Accessed May 21, 2026).

[B50] World Health Organization (2021). WHO global air quality guidelines: particulate matter (PM_2_.5 and PM10), ozone, nitrogen dioxide, sulfur dioxide and carbon monoxide. Geneva: World Health Organization. Available online at: https://www.who.int/publications/i/item/9789240034228 (Accessed June 4, 2026). 34662007

[B51] Xu B. Wang J. Li Z. Xu C. Liao Y. Hu M. . (2021). Seasonal association between viral causes of hospitalised acute lower respiratory infections and meteorological factors in China: a retrospective study. Lancet Planet. Health 5, e154–e163. doi: 10.1016/S2542-5196(20)30297-7 33713616

[B52] Yaman M. Hazar S. Yeşil E. Havan M. (2022). Detection of viral respiratory factors via multiplex PCR in newborn and pediatric patients and their distribution according to seasons. Med. Sci. Discov. 9, 586–592. doi: 10.36472/msd.v9i10.826

[B53] Yüksel Y. Bedel C. Selvi F. Zortuk O. Karanci Y. (2024). Investigating the meteorological factors influencing upper respiratory tract infections: an observational study. J. Prev. Complement. Med. 3, 167–172. doi: 10.22034/jpcm.2024.481418.1187

