# Supplementary material for: Age-stratified respiratory viral detection patterns and exploratory environmental associations in a tertiary-care hospital testing cohort in Istanbul, Türkiye
Source: Front Microbiol. 2026 Jul 16;17:1893118. doi: 10.3389/fmicb.2026.1893118 (PMC13422450; doi:10.3389/fmicb.2026.1893118)
Supplement: Supplementary file 4 [file Table_4.docx]

**Supplementary Material**

**Contents**

• Supplementary Methods. Multiplex molecular respiratory viral testing

• Supplementary Table 1. Thermal cycling conditions of the multiplex RT-PCR protocol

• Supplementary Table 2. Respiratory viral targets included in the multiplex panel

**Supplementary Methods. Multiplex molecular respiratory viral testing**

Upper respiratory tract specimens were collected as nasopharyngeal swabs and transported to the laboratory in vNAT® transfer tubes containing 2 mL of transport medium (Bioeksen R&D Technologies, Istanbul, Türkiye). Samples were processed according to the manufacturer’s recommendations before routine diagnostic testing.

Respiratory viral targets were detected using the Bio-Speedy Respiratory Tract RT-PCR MX-24S Panel (Bioeksen R&D Technologies, Istanbul, Türkiye). For multiplex reverse transcription polymerase chain reaction testing, 90 µL of patient sample from the vNAT tube was mixed with 90 µL of 2× Prime Master Mix, resulting in a total preparation volume of 180 µL. Amplification was performed on the CFX96 Real-Time PCR System (Bio-Rad, USA) according to the manufacturer’s thermal cycling protocol.

**Supplementary Table 1. Thermal cycling conditions of the multiplex RT-PCR protocol.**

| **Step** | **Temperature (°C)** | **Time** | **Cycles** |
| --- | --- | --- | --- |
| **Initial steps** |  |  | **1** |
| Enzyme activation | 52 | 3 min |  |
| Pre-denaturation | 95 | 10 sec |  |
| **Touchdown phase** |  |  | **12** |
| Denaturation | 95 | 1 sec |  |
| Annealing and extension | 67 → 56* | 15 sec |  |
| **Amplification phase** |  |  | **30** |
| Denaturation | 95 | 1 sec |  |
| Annealing and extension** | 55 | 15 sec |  |

* Temperature decreased by 1°C per cycle during the touchdown phase.

** Fluorescence signal detection (FAM, HEX, ROX, Cy5) was performed during this step.

**Supplementary Table 2. Respiratory viral targets included in the multiplex panel.**

| **Target group** | **Respiratory viral targets** |
| --- | --- |
| Coronaviruses | SARS-CoV-2; seasonal human coronaviruses (HCoV-229E, HCoV-OC43, HCoV-NL63, and HCoV-HKU1) |
| Influenza viruses | Influenza A virus (IAV); influenza B virus (IBV) |
| Parainfluenza viruses | Human parainfluenza virus types 1–4 (HPIV-1, HPIV-2, HPIV-3, HPIV-4) |
| Other respiratory viruses | Human metapneumovirus (hMPV); adenovirus (AdV); human bocavirus (HBoV); enterovirus/rhinovirus (E-RV); human parechovirus (HPeV); respiratory syncytial virus A/B (RSV A/B) |
